# Factors Related to the Behavior of People Who Have Never Used the Internet for Voluntary Reasons: Cross-Sectional Survey Study

**DOI:** 10.2196/20453

**Published:** 2020-11-11

**Authors:** Keeho Park

**Affiliations:** 1 Graduate School of Cancer Science and Policy National Cancer Center Goyang Republic of Korea

**Keywords:** internet, use, voluntary, factor, information and communications technology, digital divide

## Abstract

**Background:**

If there are people who do not want to use the internet despite having the circumstances and conditions for using it, another policy consideration will be needed.

**Objective:**

The purpose of this study was to explore the factors related to the behavior of people who do not voluntarily use the internet.

**Methods:**

A cross-sectional survey was conducted in 2018. It used a proportional quota random sampling design to select a representative sample of Koreans. Accordingly, 6150 participants were included in the study. Multiple logistic regression methods were used to explore the predicting factors of the act of voluntarily not using the internet.

**Results:**

Age, education level, bonding and bridging social capitals, and daily life satisfaction for health status were found to be factors related to the behavior of not voluntarily using the internet. However, gender, household income, occupation, family size, and community type were not related to voluntary nonuse of the internet.

**Conclusions:**

It was found that sociodemographic factors, such as age and education level, which are difficult to modify, along with psychosocial factors located deeper than the visible living conditions, such as social capital and life satisfaction, are involved in voluntary internet nonuse. These results also suggest that it is not desirable to proceed with policies related to information and communications technology on a separate track, but rather that they should be comprehensively approached with other social policies that design various social interventions in order to enhance equity within the society.

## Introduction

The internet is now an essential part of life. However, this may not be the case in countries where internet infrastructure is not available nationwide or in countries where there are few people who can pay for internet services. Even if people do not live in such a country, there may be variations in the available digital life within the country or community from a digital divide caused by various factors.

In countries with advanced information and communications technology (ICT), attempts are underway to actively utilize these technologies for providing social services [1]. Notably, even if internet access is improved for those who are targeted for social welfare services, such efforts will not be effective unless the fundamental social vulnerabilities that interfere with the intention to use the internet are addressed.

When it comes to the internet, its use is very common in South Korea, where there is a good environment that allows it to be effective. South Korea is the 12th largest country in the world according to gross domestic product (GDP) [2]. As of 2018, the internet access rate was the highest in South Korea at 99.5% (including wired and wireless), followed by Iceland (99.2%), the Netherlands (98.0%), Norway (96.0%), the United Kingdom (94.8%), Germany (94.8%), and Finland (94.3%) [3]. Studies of people who do not use the internet on a voluntary basis, even though they live in a country with well-developed ICT, may be useful policy material for not only those countries but also ones that currently lack ICT.

When discussing the social problem of internet use, researchers have mainly talked about vulnerability related to various factors on the premise that the internet should be available to anyone if they want it. Nevertheless, if there are people who do not want to use the internet despite having the circumstances and conditions for using it, another policy consideration will be needed. According to the Pew Research Center’s report on the five factors related to internet nonuse, 15% of Americans do not use the internet and 92% of adults who do not use the internet said that they were not interested in using the internet or email in the future [4]. In the survey, the following factors were related to digital divide: age, income and educational attainment, community type, disability, and Spanish-speaking preference. Previous studies that analyzed factors related to internet use have reported age, disability, income, and education level as related factors [5-8].

However, it can be said that the factors that impede the use of the internet and the factors related to the nonuse of the internet due to voluntary intention have different meanings. As shown in the findings above, it is likely that people who are not willing to use the internet, especially those who have various conditions to use the internet, may have different contexts in the matter of digital divide.

This attitude toward internet use may be a sign of a more fundamental social vulnerability and not just about the internet. For those who have uncommon behaviors for voluntary reasons like this, it is necessary to examine the social and psychological situations they face.

High subjective well-being is associated with many desirable outcomes [9], such as positive development among young adults [10], healthier and longer lives [10], and democratic attitudes [11]. In a longitudinal study [12], the authors suggested that life satisfaction is prospectively associated with the occurrence of several major events in work and family life. Because life satisfaction is a trait-like construct [12,13], persons with higher satisfaction are more likable and more socially active [14] and are therefore more likely to have satisfying social relationships [12]. Additionally, there are research findings suggesting that life satisfaction motivates a greater willingness to engage in preventive health care, and these behaviors have been reported [15-18]. As such, life satisfaction not only has meaning as a result of certain life events, but also is a factor influencing an individual’s attitude and behavior.

Social capital is a source of empowerment [19,20] and interacts with policy implementation [21]. Social capital can affect individuals’ risk for negative health outcomes and their engagement in risk behaviors [22]. Putnam [23] has explained the distinction between bridging and bonding social capitals. Bonding social capital is inward looking and tends to reinforce exclusive identities and homogeneous groups. On the other hand, bridging social capital is outward looking and encompasses people across diverse social cleavages. A study that was conducted to explore the relationship between social capital and willingness to join a community-based health insurance program found that social capital was a key determinant of willingness to join the program, and that people with a high level of bonding capital would be reluctant to cooperate with others [24]. In a study that explored the literature on social capital, human capital, and social learning theory, the author raised the hypothesis that the decline in social capital is negatively influencing recent college graduates’ formation of soft skills. It was also suggested that college students gain the cultural and behavioral information and sensitivity they need to learn soft skills through the process of building social capital [25]. Additionally, social capital has a significant effect on information-related behaviors, particularly on the choice of source [26].

Thus, it can be assumed that regardless of the abundance of material resources, a poor level of subjective well-being or social capital can lead to a vicious cycle that inhibits universal and positive social behaviors. This study aimed to find out how life satisfaction and social capital act on voluntary nonusers of the internet, who live in South Korea.

## Methods

### Study Participants

We used data from the 2018 Survey on the Actual Condition of the Digital Divide conducted in South Korea. Since 2002, this survey has been annually conducted by the Ministry of Science and ICT along with the National Information Society Agency (NIA). It is done in order to check the annual performance of the digital information disparity policy through time-series research and analysis, as well as to provide the basic data necessary for future effective policy implementation. The survey consists of the following two parts: (1) digital informatization level and (2) attitudes toward information use. The data for each year of the survey were collected via a cross-sectional and nationally representative survey using a multistage stratified sampling design. After obtaining informed consent from participants, the health interview was performed by trained interviewers of the research corporation Kantar Korea. The survey was approved by the institutional review board at the NIA.

This survey can also be found in another research paper [27]. The survey data for 2018 included information for 7000 individuals. After the exclusion of those who were aged ≤18 years, 6150 participants remained in the study.

### Assessment

Age, gender, education, occupation, household income, family size, and community type were included as sociodemographic variables (Table 1). The variables were categorized as follows: age (19-29, 30-39, 40-49, 50-59, or ≥60 years); education level (elementary school or lower, middle school, high school, or college graduate or higher); occupation (office/sales/technical/professional, agriculture/forestry/fishery, simple labor, housewife, student, or not employed); average monthly household income (≤0.99, 1-1.99, 2-2.99, 3-3.99, 4-4.99, 5-5.99, or ≥6.0 million Korean won [KRW]/month) (1 KRW = 0.00089 USD); family size (single-person household or multi‑person household); and community type (urban or rural). In the questionnaire for disability, whether the respondent was a registered disabled person was investigated.

To investigate the respondents’ internet usage, the question, “When was the last time you recently used the internet?” was asked. The factors involved with this question included the use of PCs, mobile phones, tablet PCs, Internet Protocol televisions, etc, regardless of location. In response, respondents were asked to choose one of the following: “Within the last month,” “I have not used the internet for more than a month,” and “I have never used the internet.” The respondents were asked to choose one of the following reasons why they did not use the internet: “I am willing to use it, but I am not able to because I am not able to use it (expensive rates, no digital devices to use, physical disabilities, difficulties in how to use it, etc)” or “I can use it, but I do not feel the need to use it, so I do not use it.” For those who responded “I can use it, but I do not feel the need to use it, so I do not use it,” the following additional question was asked: “What is the main reason you do not use the internet?.” In response, respondents were asked to choose one of the following: “Because there is no inconvenience without using the internet,” “I have a negative thought about using the internet (wasting time, not very helpful, etc),” “Because of the reluctance to learn and use new things,” “Because most people who are close to me do not use the internet,” and “I am concerned about spam mail, personal information leakage, illegal information distribution, internet addiction, etc.”

Social capital was measured using the Internet Social Capital Scale (ISCS) [28], which was developed based on the conceptualization of bonding and bridging social capital by Putnam [23]. The ISCS consists of 10 items for bonding social capital and 10 items for bridging social capital. A 4-point Likert scale instead of the original 5-point scale was used for each of the measurement items, ranging from 1 (strongly disagree) to 4 (strongly agree). The Cronbach alpha coefficient for this scale was .898.

In order to investigate satisfaction in various aspects of daily life, satisfaction was examined in the following eight areas: leisure and cultural life, economic conditions, social activities (community, gathering, community participation, etc), interpersonal relationships, family relationships, what I do (such as academic or work activities), health status, and politics or government policy. The daily life satisfaction was measured by having respondents choose “not satisfied at all,” “unsatisfied,” “satisfied,” or “very satisfied” as an answer to the question, “How satisfied are you with the items below in your daily life?.” The Cronbach alpha coefficient for this scale was .797.

Life satisfaction was assessed using the Satisfaction with Life Scale (SWLS), which was developed by Diener et al [29]. The SWLS consists of five items that measure satisfaction with one’s life. Participants were asked to rate each item on a 5-point Likert scale ranging from 1 (strongly disagree) to 7 (strongly agree), and the total score could range from 5 to 35. The total scores of the scale can be interpreted as “extremely dissatisfied” (5-9), “dissatisfied” (10-14), “slightly below average in life satisfaction” (15-19), “average score” (20-24), “high score” (25-29), or “very high score (highly satisfied with life)” (30-35). The Korean version was obtained from the public database of the original author [30]. The Cronbach alpha coefficient for this scale was .887.

**Table 1:** Characteristics of the sample (N=6150).

Characteristic		Value, n (%) or mean (SD)
**Gender (missing: 99, 1.61%)**		
	Male	3075 (50.82)
	Female	2976 (49.18)
**Age (years)**		
	19-29	1033 (16.80)
	30-39	864 (14.05)
	40-49	1015 (16.50)
	50-59	1531 (24.89)
	≥60	1707 (27.76)
**Education**		
	Elementary school or lower	452 (7.35)
	Middle school	725 (11.79)
	High school	3000 (48.78)
	College or higher	1973 (32.08)
**Household income (million KRW^a^/month)**		
	≤0.99	226 (3.67)
	1-1.99	706 (11.48)
	2-2.99	1025 (16.67)
	3-3.99	1496 (24.33)
	4-5.99	2254 (36.65)
	≥6	432 (7.02)
**Occupation**		
	Office, sales, technical, or professional	3935 (63.98)
	Agriculture, forestry, or fishery	175 (2.85)
	Simple labor	189 (3.07)
	Student	390 (6.34)
	Housewife	1107 (18.00)
	Not employed	354 (5.76)
**Family size**		
	Single-person household	516 (8.39)
	Multi-person household	5634 (91.61)
**Community type**		
	Urban	4642 (75.48)
	Rural	1508 (24.52)
Disability (yes)		70 (1.14)
**Internet usage**		
	Voluntary nonuser	346 (5.63)
	Others	5804 (94.37)
Bonding social capital score, mean (SD)		27.09 (4.61)
Bridging social capital score, mean (SD)		27.85 (5.44)
**Satisfaction in various aspects of daily life**		
	Leisure and cultural life (satisfied)	4442 (72.23)
	Economic conditions (satisfied)	3592 (58.41)
	Social activities (community, gathering, community participation, etc; satisfied)	4099 (66.65)
	Interpersonal relationships (satisfied)	5149 (83.72)
	Family relationships (satisfied)	5516 (89.69)
	What I do (such as academic and work activities; satisfied)	4439 (72.18)
	Health status (satisfied)	4602 (74.83)
	Politics or government policy (satisfied)	2999 (48.76)
**Life satisfaction**		
	Equal or higher than average	4079 (66.33)
	Lower than average	2071 (33.67)

^a^KRW: Korean won.

### Statistical Analysis

In this study, a voluntary nonuser of the internet was defined as a person who answered in the questionnaire that he or she had never used the internet and responded that he or she had the condition or ability to use the internet but did not feel the need to use it.

The daily life satisfaction categories of “not satisfied at all” and “unsatisfied” were grouped as “not satisfied,” and the categories of “satisfied” and “very satisfied” were grouped as “satisfied.” Accordingly, they were used as binary variables for the chi-square test and multiple logistic regression.

The SWLS categories of “extremely dissatisfied” (5-9), “dissatisfied” (10-14), and “slightly below average in life satisfaction” (15-19) were categorized as “lower than average,” and the categories of “average score” (20-24), “high score” (25-29), and “very high score or highly satisfied with life” (30-35) were categorized as “equal or higher than average.” Accordingly, they were used as binary variables for the chi-square test and multiple logistic regression.

To explore crude associations between potentially explanatory variables, such as sociodemographic variables, disability, daily life satisfaction, life satisfaction, and the act of voluntarily not using the internet, the Pearson chi-square test was conducted. A Student *t* test was used to compare the mean of bonding social capital and bridging social capital between the two groups based on whether individuals were not voluntarily using the internet. Multiple logistic regression explored the predicting factors of the act of voluntarily not using the internet. Variables with a significance level of *P*<.05 in the bivariate analysis were entered into the multivariate model. The analysis was performed using IBM SPSS Version 16 software (IBM Corp).

### Ethics Approval

This study was approved by the NIA Institutional Review Board. All of the participants provided written informed consent for the survey. The funding source had no role in writing or submitting this research paper.

## Results

Table 1 shows the general characteristics of the participants. A total of 346 respondents (N=6150, 5.63%) represented voluntary nonusers of the internet. In most cases, the education level was above middle school. Overall, 69.90% (4299/6150) of respondents were employed and 18.00% (1107/6150) were housewives. Moreover, 24.52% (1508/6150) of the respondents were living in rural areas and 1.14% (70/6150) were registered as being disabled.

The mean score of bonding social capital was similar to that of bridging social capital. In terms of satisfaction in various aspects of daily life, the fraction of those satisfied was the highest for family relationships (5516/6150, 89.69%) and the lowest for politics or government policies (2999/6150, 48.76%). Regarding the SWLS score, 66.33% (4079/6150) of respondents had an “equal or higher than average” score.

For the question, “What is the main reason you do not use the internet?,” the frequencies of responses were as follows: “Because there is no inconvenience without using the internet,” 82.1%; “I have a negative thought about using the internet (wasting time, not very helpful, etc),” 5.5%; “Because of the reluctance to learn and use new things,” 7.8%; “Because most people who are close to me do not use the internet,” 3.2%; “I am concerned about spam mail, personal information leakage, illegal information distribution, internet addiction, etc,” 1.2%; and “other,” 0.2%.

When the association between various factors and internet usage was assessed, the variables that showed statistical significance were gender, age, education, household income, occupation, family size, community type, disability, bonding social capital, bridging social capital, daily life satisfaction (in every aspect), and life satisfaction (SWLS) (Table 2).

Although there are limitations in interpretation because the approach is a univariate analysis, the percentage of people who did not voluntarily use the internet was higher among women, elderly people, less educated people, people with low income, unemployed people, agriculture/forestry/ fishery workers, simple laborers, housewives, people in single-person households, and disabled people.

For people who did not voluntarily use the internet, the bonding social capital and bridging social capital scores were lower and the score difference was greater for bridging social capital. Among those who were not satisfied in various aspects of life, the percentage of people who did not voluntarily use the internet was higher. This trend was the same in the case of life satisfaction measured by the SWLS.

A multivariate logistic regression analysis was conducted to explore the predictors of the behavior of not voluntarily using the internet (Table 3). We reviewed the odds ratio (OR) and CI of each calculation. Age, education level, bonding and bridging social capitals, and daily life satisfaction for health status were found to be factors related to the behavior of not voluntarily using the internet. However, gender, household income, occupation, family size, and community type were not related to voluntary internet nonuse.

As age increased by 1 year, the likelihood of not voluntarily using the internet increased 1.14 times (OR 1.14, 95% CI 1.12-1.17). A higher level of education was associated with less likelihood that people would not voluntarily use the internet. The likelihood that a person with a higher education level than a university degree would not voluntarily use the internet was 0.14 times the likelihood for a person with a lower education level than middle school (OR 0.14, 95% CI 0.05-0.45). In the case of social capital, bonding social capital and bridging social capital showed different directions for internet use behavior. As the score of bonding social capital increased by 1, the likelihood of not voluntarily using the internet increased by 1.04 times (OR 1.04, 95% CI 1.01-1.08). On the other hand, the likelihood of not using the internet voluntarily decreased by 0.93 times as the score of bridging social capital increased by 1 (OR 0.93, 95% CI 0.91-0.96). Among the many aspects of everyday life, those who were not satisfied with their health status were 1.38 times more likely to not voluntarily use the internet than those who were satisfied (OR 1.38, 95% CI 1.01-1.87).

**Table 2:** Association between various factors and life satisfaction.

Characteristic			Internet usage, n (%) or mean (SD)			*P* value
			Voluntary nonuser	Others		
**Gender**					<.001^a^	
	Male (n=3075)		114 (3.7)	2961 (96.3)		
	Female (n=2976)		223 (7.5)	2753 (92.5)		
**Age** (years)					<.001^a^	
	19-29 (n=1033)		0 (0.0)	1033 (100.0)		
	30-39 (n=864)		0 (0.0)	864 (100.0)		
	40-49 (n=1015)		0 (0.0)	1015 (100.0)		
	50-59 (n=1531)		12 (0.8)	1519 (99.2)		
	≥60 (n=1707)		334 (19.6)	1373 (80.4)		
**Education**					<.001^a^	
	Elementary school or lower (n=452)		160 (35.4)	292 (64.6)		
	Middle school (n=725)		121 (16.7)	604 (83.3)		
	High school (n=3000)		61 (2.0)	2939 (98.0)		
	College or higher (n=1973)		4 (0.2)	1969 (99.8)		
**Household income (million KRW^b^/month)**					<.001^a^	
	≤0.99 (n=226)		85 (37.6)	141 (62.4)		
	1-1.99 (n=706)		141 (20.0)	565 (80.0)		
	2-2.99 (n=1025)		61 (6.0)	964 (94.0)		
	3-3.99 (n=1496)		33 (2.2)	1463 (97.8)		
	4-5.99 (n=2254)		22 (1.0)	2232 (99.0)		
	≥6 (n=432)		4 (0.9)	428 (99.1)		
**Occupation**					<.001^a^	
	Office, sales, technical, or professional (n=3935)		78 (2.0)	3857 (98.0)		
	Agriculture, forestry, fishery (n=175)		24 (13.7)	151 (86.3)		
	Simple labor (n=189)		19 (10.1)	170 (89.9)		
	Student (n=390)		0 (0.0)	390 (100.0)		
	Housewife (n=1107)		139 (12.6)	968 (87.4)		
	Not employed (n=354)		86 (24.3)	268 (75.7)		
**Family size**					<.001^a^	
	Single-person household (n=516)		88 (17.1)	428 (82.9)		
	Multi-person household (n=5634)		258 (4.6)	5376 (95.4)		
**Community type**					.01^a^	
	Urban (n=4642)		241 (5.2)	4401 (94.8)		
	Rural (n=1508)		105 (7.0)	1403 (93.0)		
**Disability**					<.001^a^	
	Yes (n=70)		12 (17.1)	58 (82.9)		
	No (n=6080)		334 (5.5)	5746 (94.5)		
Bonding social capital score, mean (SD)			25.07 (4.81)	27.21 (4.57)	<.001^c^	
Bridging social capital score, mean (SD)			23.25 (6.17)	28.12 (5.27)	<.001^c^	
**Satisfaction in various aspects of daily life**						
	**Leisure and cultural life**				<.001^a^	
		Satisfied (n=4442)	149 (3.4)	4293 (96.6)		
		Dissatisfied (n=1708)	197 (11.5)	1511 (88.5)		
	**Economic conditions**				<.001^a^	
		Satisfied (n=3592)	111 (3.1)	3481 (96.9)		
		Dissatisfied (n=2558)	235 (9.2)	2323 (90.8)		
	**Social activities (community, gathering, community participation, etc)**				<.001^a^	
		Satisfied (n=4099)	163 (4.0)	3936 (96.0)		
		Dissatisfied (n=2051)	183 (8.9)	1868 (91.1)		
	**Interpersonal relationships**				<.001^a^	
		Satisfied (n=5149)	244 (4.7)	4905 (95.3)		
		Dissatisfied (n=1001)	102 (10.2)	899 (89.8)		
	**Family relationships**				<.001^a^	
		Satisfied (n=5516)	289 (5.2)	5227 (94.8)		
		Dissatisfied (n=634)	57 (9.0)	577 (91.0)		
	**What I do (such as academic and work activities)**				<.001^a^	
		Satisfied (n=4439)	172 (3.9)	4267 (96.1)		
		Dissatisfied (n=1711)	174 (10.2)	1537 (89.8)		
	**Health status**				<.001^a^	
		Satisfied (n=4602)	139 (3.0)	4463 (97.0)		
		Dissatisfied (n=1548)	207 (13.4)	1341 (86.6)		
	**Politics or government policy**				<.001^a^	
		Satisfied (n=2999)	104 (3.5)	2895 (96.5)		
		Dissatisfied (n=3151)	242 (7.7)	2909 (92.3)		
**Life satisfaction**					<.001^a^	
	Equal or higher than average (n=4079)		159 (3.9)	3920 (96.1)		
	Lower than average (n=2071)		187 (9.0)	1884 (91.0)		

^a^*P* value determined using the Pearson chi-square test.

^b^KRW: Korean won.

^c^*P* value determined using Student *t* test.

**Table 3:** Multiple logistic regression results examining the factors associated with the act of voluntarily not using the internet.

Characteristic			Voluntary nonusers		
			Adjusted OR^a^	95% CI	
Gender (female)			1.14	0.78-1.66	
Age (continuous)			1.14	1.12-1.17	
**Education**					
	Elementary school or lower		Reference	N/A^b^	
	Middle school		0.82	0.59-1.15	
	High school		0.43	0.28-0.66	
	College or higher		0.14	0.05-0.45	
**Household income (million KRW^c^/month)**					
	≤0.99		Reference	N/A	
	1-1.99		0.85	0.55-1.33	
	2-2.99		0.73	0.43-1.24	
	3-3.99		0.73	0.39-1.36	
	4-5.99		0.58	0.29-1.15	
	≥6		0.64	0.20-2.08	
**Occupation**					
	Office, sales, technical, or professional		Reference	N/A	
	Agriculture, forestry, fishery		1.06	0.57-1.97	
	Simple labor		1.01	0.56-1.83	
	Student		—^d,e^	—^d,e^	
	Housewife		1.20	0.80-1.82	
	Not employed		1.25	0.81-1.94	
**Family size**					
	Single-person household		Reference	N/A	
	Multi-person household		0.88	0.58-1.33	
**Community type**					
	Urban		Reference	N/A	
	Rural		0.91	0.66-1.27	
**Disability**					
	No		Reference	N/A	
	Yes		0.76	0.35-1.66	
Bonding social capital score (continuous)			1.04	1.01-1.08	
Bridging social capital score (continuous)			0.93	0.91-0.96	
**Satisfaction in various aspects of daily life**					
	**Leisure and cultural life**				
		Satisfied	Reference	N/A	
		Dissatisfied	1.22	0.88-1.68	
	**Economic conditions**				
		Satisfied	Reference	N/A	
		Dissatisfied	1.16	0.82-1.64	
	**Social activities (community, gathering, community participation, etc)**				
		Satisfied	Reference	N/A	
		Dissatisfied	0.85	0.62-1.17	
	**Interpersonal relationships**				
		Satisfied	Reference	N/A	
		Dissatisfied	1.10	0.77-1.56	
	**Family relationships**				
		Satisfied	Reference	N/A	
		Dissatisfied	1.00	0.68-1.47	
	**What I do (such as academic and work activities)**				
		Satisfied	Reference	N/A	
		Dissatisfied	0.85	0.62-1.16	
	**Health status**				
		Satisfied	Reference	N/A	
		Dissatisfied	1.38	1.01-1.87	
	**Politics or government policy**				
		Satisfied	Reference	N/A	
		Dissatisfied	0.96	0.69-1.33	
**Life satisfaction**					
	Equal or higher than average		Reference	N/A	
	Lower than average		1.20	0.88-1.63	

^a^OR: odds ratio.

^b^N/A: not applicable.

^c^KRW: Korean won.

^d^For students, the number of voluntary internet nonusers is zero.

^e^Not available.

## Discussion

The purpose of this study was to explore the factors related to the behavior of people who do not voluntarily use the internet, regardless of conditions. As factors related to such voluntary behavior regardless of conditions, age and education level, which were previously reported to be related to internet use, have been reaffirmed. However, household income and disability were not related to the act of voluntarily not using the internet regardless of the circumstances. These results suggest that spontaneity related to the nonuse of the internet is not related to income or disability; yet, spontaneity is affected when age increases or the level of education is low. These results mean that countries or communities that promote an informatized society should help the elderly or less educated to easily understand the benefits of the internet that they have never experienced and increase their ability to use it. Interestingly, it can be seen that social capital exerts different influences on the voluntary nonuse of the internet, depending on its attributes. The finding that a higher bonding social capital is associated with higher voluntary nonuse of the internet suggests that the unmet needs are somewhat being met by the support or help of others, even if the individuals do not use the internet in their own group. On the other hand, the finding that a higher bridging social capital is associated with lower voluntary internet nonuse can be seen as a natural result reflecting the interest and willingness in communicating with other groups outside of their own group. As a strategy for voluntary internet nonusers who are reluctant to use the internet, it can be considered to promote campaigns targeting existing internet users in order to ensure that changes in attitudes and behaviors of people with low bridging social capital can occur owing to the influence of people with high levels of ICT use in the groups.

In terms of life satisfaction, only satisfaction with health status was related to voluntary internet nonuse. It may not be something to be taken lightly that a person who is not satisfied with his or her health does not want to use the internet. Those who are not satisfied with their health owing to poor objective or subjective health conditions may find various communication or participation activities, and internet use annoying. For these people, quality of life can worsen by entering a vicious circle.

In this study, it was found that sociodemographic factors, such as age and education level, which are difficult to modify, and psychosocial factors located deeper than the visible living conditions, such as social capital and life satisfaction, are involved in voluntary internet nonuse. These results also suggest that it is not desirable to proceed with ICT-related policies on a separate track, but rather that they should be comprehensively approached together with other social policies that design various social interventions in order to enhance equity within the society. The results of this study suggest that in order to fully extract the roots of the weed called digital divide, it is necessary to consider the sociopsychological soil that can sustain the roots. The recent COVID-19 pandemic of 2019 has led many countries to consider the activation of telemedicine or telehealth [31,32]. Compared with the rapidly changing global health care situation and the development of ICT, it is time to carefully review whether the social welfare infrastructure of each country can keep pace.

This study has several strengths. First, it used nationally representative data gathered by a professional research firm using random probability allocation. Second, relatively diverse variables related to internet usage were investigated and used for multivariate analysis. Third, this study was conducted in a country with well‑equipped ICT infrastructure. This research environment is suitable for investigating relevant factors for voluntary internet users regardless of conditions, and it can provide useful information to many countries that have built or are building a similar level of ICT infrastructure. On the other hand, this study has some limitations. This study is a cross-sectional study (not a longitudinal one), and so, there are limitations regarding the interpretation of the study results. Moreover, no detailed psychological investigations were carried out to determine which psychological mechanisms exist between low satisfaction with health status and voluntary internet nonuse. In the future, it seems necessary to produce more thorough evidence utilizing community intervention studies targeting the vulnerable groups of the internet, including voluntary internet nonusers.

## Abbreviations

ICT: information and communications technology
ISCS: Internet Social Capital Scale
NIA: National Information Society Agency
OR: odds ratio
SWLS: Satisfaction with Life Scale
